# Reflexivity and humility evoke a transformable methodology in a post disaster context

**DOI:** 10.1080/21642850.2020.1862661

**Published:** 2021-01-28

**Authors:** Mariana T. Guzzardo, Irina L. G. Todorova, Alina Engelman, Manuela Polidoro Lima, Evelyn Dean-Olmsted, Rosa E. Guzzardo Tamargo

**Affiliations:** a Department of Human Development and Women's Studies, California State University, East Bay, Hayward, CA, USA; b Applied Psychology, Northeastern University, Boston, MA, USA; c Health Sciences, California State University, East Bay, Hayward, CA, USA; d Psychology, Pontifícia Universidade Católica do Rio Grande do Sul, Porto Alegre, Brazil; e Anthropology, University of Puerto Rico, Río Piedras, Puerto Rico; f Linguistics and Hispanic Studies, University of Puerto Rico, Río Piedras, Puerto Rico

**Keywords:** Disaster, qualitative, Puerto Rico, disabilities, interpretative phenomenological analysis, relational inquiry, decolonial methodologies

## Abstract

**Objective:**

The process of reflexivity is used to critically examine the experience of conducting qualitative research with functionally diverse older adults in a post disaster context.

**Methods:**

The design of the study began with an interpretative phenomenological framework, using in-depth interviews. Fifteen individuals with functional and access needs living in Puerto Rico were interviewed regarding their experiences after Hurricane María of 2017.

**Findings:**

In the field, it was necessary to expand the initial design, and adjust to participants’ preferences and needs, as well as situational characteristics, without compromising ethical standards of practice. The methodology transformed because of the need for flexibility requiring humility from the researchers. A more relational form of inquiry was warranted, which acknowledged the intersubjectivity of human experience. This entailed adapting to community involvement, building rapport with community leaders functioning as gatekeepers, and integrating family or friends in interviews.

**Discussion:**

The reflexive approach allowed for a better understanding of the researcher’s positionalities and how they influence the ability or inability to develop trust (e.g. insider/ outsider status, Puerto Rican/ US, with functional and access needs/ without functional and access needs).

**Conclusions:**

Given the shift toward relational inquiry and due to the challenges faced while carrying out the study, we suggest that post-disaster qualitative research would benefit from further including principles of indigenous decolonizing methodologies, which can be incorporated into studies using interpretative phenomenological analysis.

## Introduction

In this paper, I^1^ take a reflexive approach to consider the lessons learned during my time in the field, investigating the experiences of older Puerto Ricans with functional and access needs after Hurricane María struck the archipelago of Puerto Rico in September of 2017. Reflexivity is essential to the process of conducting qualitative and quantitative research, since it allows for the necessary introspection and examination of one’s own positionalities in relation to the participants of the research project, when assuming the role of researcher (Indah, 2018). Additionally, reflexivity contributes to a retrospection about the process and enables engagement in critical evaluation that, in our case, entailed expanding our Interpretative Phenomenological Analysis (IPA) methodology.

In order to describe this methodological process, this paper has a nontraditional structure. It is our hope that the reader can perceive it as a story that retells the ways in which our research evolved; we feel that this story-telling style is quite compatible with the reflexive approach. The intent is to narrate about the process of the research project through the chronological occurrence of events while explaining how our thinking developed and our methods transformed. First, we provide some detail about where this project started, as well as the process of designing the study with consideration of our initial research objectives. In this section, I explore my perspective as a Puerto Rican and as an academic. Second, we narrate the emergence of this research in Puerto Rico (PR) and this unique context given its neocolonial status and particular hardships. Third, we write about carrying out the study and how the reflexive approach necessitated humility which allowed space for a transformable methodology, including the following: adapting to the challenges surrounding reaching and recruiting participants, positioning and questioning of insider/outsider status while in the field, and making changes to the design and its implementation. Methods transformed into a more relational form of inquiry that acknowledged the intersubjectivity of human experience, that is, the influence of the relationship between the participant and the researcher as well as the interaction with other people in the participant’s environment. Lastly, we discuss what we learned through this process, and make suggestions for future post-disaster qualitative research, including the overall critical importance of flexibility, sensitivity, and humility when conducting this work. The content of the paper builds toward the final suggestion for research in post-disaster contexts and in PR: to use the principles of indigenous decolonizing methodologies, which entail a focus on the participants’ goals and perspectives and a more active participant role in decisions pertaining to the research design, data collection, and use of the findings (Braun, Browne, Ka’opua, Kim, & Mokuau, 2014; Keikelame & Swartz, 2019; Tuhiwai Smith, 2012; Zavala, 2013).

The reflective approach in this paper, when considering the dilemmas faced during the research process, contributes to the critical evaluation of our praxis. Indah (2018) explains that it is through this reflexivity that we visibilize ‘the fieldwork’s influence on the researcher, as well as the researcher’s influence on the fieldwork’ (p. 800). As part of this reflection, we considered the adaptation to situational issues that arise, as well as adjustment to the preferences and circumstances of the people involved in the research.

At the start of the century, Chamberlain (2000) shared concerns about overemphasizing methodology, particularly in qualitative research in health psychology, having to do with ‘over-ardent concern with methodology, a privileging of methods over other considerations’ (p. 287). Chamberlain (2000) describes some of the potential consequences of such strict prioritization of methods, which we found to be certainly relevant in our work in the post-disaster context in PR. While a research project should always keep to ethical standards for human subject research, there can also be some level of transformability in the design, particularly in the context of post-disaster research, which poses special considerations.

It is sometimes necessary to not let predetermined decisions about the research become detrimental to its ultimate goals, which are to ensure self-determination and dignity for the people involved in our study and to understand their perspective based on what they want to reveal to us. The protocol established by the method may impose restrictions that hinder our need for sensitivity and flexibility when working with older individuals who experience functional diversity^2^ and have survived a disaster. In the post disaster context of Hurricane María’s effect on PR, the methodology included being reflexive and open to redefining and reconceptualizing this project within the challenges and the context of loss and suffering that emerged.

## Background

### Qualitative disaster research and related concepts

When interviewing individuals who have been through a disaster or traumatic event, it is critical to consider Bourdieu’s (1996) conceptualization of the ‘social relation’ between the interviewer and the interviewee. He explains that there is a ‘social asymmetry’ within this research relationship due to the interviewer’s social or linguistic capital. This can potentially lead to a ‘symbolic violence,’ partly comprised by the interviewer’s ‘intrusion’ or effects on the interviewee and their/ her/ his responses. Therefore, research entails becoming aware of our presuppositions through reflexivity: ‘It is to attempt to bring out the representation the respondent has of the situation, of the survey in general, of the particular relationship in which it is taking place, of the ends it is pursuing, and to make explicit the reasons which led her to agree to take part in the exchange’ (p. 18). In other words, the qualitative researcher accepts, acknowledges, and analyzes the inevitability of their own presuppositions, the construction of knowledge in the research process, and its effects in the process of attempting to understand the interviewee. By engaging in this process of reflexivity, the researcher can contribute to reducing the symbolic violence in the interview relationship.

Reflexivity has also been considered thoroughly as a tool for achieving ethical practice in qualitative research (Bowtell, Sawyer, Aroni, Green, & Duncan, 2013; Case, 2017; Guillemin & Gillam, 2004; Reid, Brown, Smith, Cope, & Jamieson, 2018). Guillemin and Gillam (2004) explain what being reflexive entails: ‘a continuous process of critical scrutiny and interpretation, not just in relation to the research methods and the data but also to the researcher, participants, and the research context’ (p. 275). This includes researchers considering and analyzing their positionalities, and influence on the research. In this definition researchers are not only encouraged to be reflexive about the knowledge produced by the research (e.g. epistemology) but also about the interpersonal and ethical aspects of the research process. Reflexivity can help researchers consider their own implicit biases (Case, 2017) and can lead to ethical mindfulness, particularly in the context of sensitive moments (ethical dilemmas, emotional safety procedures and practices; Bowtell et al., 2013). Given the emotional and psychological impact of disasters (Phillips, 2014), considering ethics in disaster research is crucial, which can include forming partnerships with local researchers in the community where the research takes place, as well as considering a priori the ethical dilemmas that may arise.

Qualitative methods constitute the majority of approaches within the field of disaster studies (Donner & Diaz, 2018) and ‘the nature of disaster phenomena aligns well with the epistemological and ontological assumptions of the qualitative approach’ (p. 296). In fact, Donner and Diaz (2018) explain that qualitative design is ideal because of its flexibility within unpredictable environments and because it can provide rich insights into complex human behavior and interpersonal relationships within the context of a disaster. Additionally, there are some specific characteristics of qualitative disaster research that entail interviews (Donner & Diaz, 2018; Phillips, 2014) which we’d like to highlight: (1) doing the research as soon as possible after a disaster is a key element of disaster research that seeks to understand individuals’ experiences during and in the immediate aftermath of the disaster, in an effort to document experiences when they are fresh in individuals’ minds, rather than based recollection; (2) in order to elicit meaningful data, participants should feel comfortable and the interviewer must develop a good rapport and interpersonal connection with participants; (3) researchers must be familiar with the language and culture where the disaster took place; (4) finding a place that is quiet and conducive to obtaining useful data may be quite difficult or impossible; (5) access to participants may be difficult and require interaction with gatekeepers, (6) when conducting interviews as part of the data collection method, participants should be considered ‘active players in jointly producing knowledge’ (Phillips, 2014, p. 542). According to Phillips (2014), ‘disaster scholars try to capture what people who face disasters think, feel, and do. This subjective, lived experience serves as the hallmark of all qualitative studies, including those on disasters’ (p. 544).

### Organic Beginnings of a study: Experiencing hurricane María as a researcher and member of the Puerto Rican diaspora

Hurricane María struck PR during my first week as an assistant professor (my first tenure-track position) at California State University, East Bay (CSUEB). I am one of many Puerto Ricans who consider themselves part of the diaspora living on the United States (US) Mainland. The excitement of the new position was completely overshadowed by being glued to any source of news that I could find about what was happening in PR. Intermittent communication with my family only contributed to the tension. Almost immediately social media groups were formed by the diaspora to share news about the hurricane and its impact on the people living on the islands of the Puerto Rican archipelago, with numerous requests for information on specific family members and friends.

During this time, I placed a Puerto Rican flag on my office door, next to an article with information about the effects of Hurricane María in PR. Within a few weeks, the flag was vandalized and crossed out, with a message suggesting that I shouldn’t display this flag, because this is ‘America.’ While this act may seem trivial to some, it was particularly heartbreaking given what was occurring in PR. To me it was an act of rejection and assertion that Puerto Ricans are invisible, even in a crisis. Given the multiple harmful messages against people of color found written on the doors of several offices in my building at the University, I know these prejudicial feelings are not unique to one person. The recent rise in racist vandalism, other hate crimes, and white supremacist propaganda on college campuses nationwide has been well-documented (see ADL, 2018; Bauman, 2018). Furthermore, the historical importance for Puerto Ricans to be able to exhibit their flag cannot be minimized. For example, there was a time in PR’s history under US rule, in which displaying a flag was considered subversive, and deemed a crime, leading to being arrested on charges of subordination (Law 52 of 1948, called Gag Law or *Ley de la Mordaza*; Duany, 2017). Therefore, the act of vandalizing and rejecting the Puerto Rican flag carries with it some historical significance.

### Historical context of Puerto Rico and the US Mainland

PR is an unincorporated territory (Duany, 2017) of the United States, sometimes referred to as ‘the oldest colony of the world’ (Indiana, 2019; Monge, 1997). Given the politically subordinate position of PR and its complicated relationship with the US, the flag is an important symbol of patriotic pride and protest. For many in the diaspora, it is a way of affirming one’s cultural or national identity. In fact, Puerto Ricans in the diaspora and those residing on the islands of the archipelago are characterized by a strong national pride for PR (Duany, 2002, 2017; Pantojas-García, 2005). Many Puerto Ricans do not call themselves ‘American,’ and instead opt for Puerto Rican, or *Borinqueños*/*Boricuas* (terms that come from the indigenous Taíno people; Duany, 2017; Picó, 2006). This patriotism toward PR as well as the affirmation of one’s identity rooted in PR as ‘homeland,’ (as opposed to the US Mainland) may relate to the ambiguous political status of PR.^3^

A few months later, a colleague, whose office door had been vandalized more aggressively than my own, invited me to speak on a panel titled ‘Community Mobilizing through Pedagogies of Healing and Liberation’ (Reyes, 2019). The participants discussed different ways of healing after suffering acts of oppression, and how one can challenge these acts through community mobilization. In my segment of the discussion, I talked about growing up in PR, the meaning of this experience for me, the challenges of PR’s colonial status, and the significance of participating in a protest led by the people of PR to demand the removal of the marine base from Vieques, one of PR’s smaller islands (McCaffrey, 2006). This had been one of the most successful protests in PR up until then.^4^ The weapons-testing that took place in the marine base on Vieques, which was perceived as a symbol of foreign military power and an imperial imposition, was considered highly destructive to the ecological system of the archipelago, as well as harmful to the people living close to the area. Research since then has provided evidence for the ongoing damage it has caused (Márquez & Porto, 2000; Sanderson et al., 2017).

These events took place during my years as a college student and, even though I had previously participated in several protests and activities in favor of PR’s independence, the most meaningful struggle for me was this protest for Vieques and the people living there. I became aware of how community organization and solidarity led to a victory against what many Puerto Ricans considered an imperial oppression. During the panel, I also commented on how Puerto Ricans are treated by the US government as second-class citizens and that, as they face the greatest crisis of recent history with Hurricane María, the Puerto Rican resilience was palpable once again through grass-roots organizing and neighborly solidarity in the recovery process. Acts of oppression by the current administrations (both in the US and on the Puerto Rican archipelago) associated with neglect and inadequate aid, were matched by community mobilization that helped people in great need to begin to ‘heal’ and recover from a disaster.

After the panel discussion, Alina Engelman, an assistant professor in Health Sciences who was in attendance, approached me and suggested we meet to discuss conducting research together on the experiences of Puerto Ricans in the aftermath of the disaster. She identifies as deaf and has expertise in the experiences of individuals with access and functional needs in emergencies, and emergency management that is inclusive of these people. Given my background in housing and services for older adults with functional diversity, and my personal interest in the subject of the aftermath of Hurricane María, we started to develop this study. Looking back, I realize that the impetus for the project emerged because it gave me the possibility to be connected to major events happening in PR. I was no different than many other Puerto Ricans in the diaspora who were also gravely concerned about the devastation left by the hurricane, and striving to connect with family or friends living there. In order to provide context for the incentive and enthusiasm for this work as a Puerto Rican, I will provide a brief background of Puerto Ricans on the US Mainland.

### Puerto Ricans in the US Mainland

Puerto Ricans in the US Mainland may include those who were born on one of the islands of the archipelago and have since moved away or those who are descendants of individuals born in PR (Acosta-Belén & Santiago, 2006; Duany, 2002). In fact, despite belonging to a family that has been living in the US for many generations, Puerto Ricans born in the US will still refer to themselves as ‘Puerto Ricans’ (Duany, 2002, 2017). There are currently 3.4 million residents on the archipelago of PR, while the number of people who identify as Puerto Rican who live on the US Mainland is even larger, according to most estimates of the last US Census (2010). Many of us in the diaspora struggle with complicated feelings for leaving PR (Morillo, 2017) recognizing how the ‘brain drain’ affects the economy and increases the daily struggles of those who remain. After I left PR, a movement called ‘yo no me quito’ (‘I won’t give up/ quit’) started around 2016, referring to young people who proclaimed themselves committed to PR and who would not ‘quit’ (give up on) PR, or move away. Some of us in the diaspora, consequently, began to feel more deeply the need to aid people living on the islands of PR, not just because of the disaster left by the hurricane, or the preceding economic crisis, but also because of the local narrative on how young people’s departure exacerbated problems in PR. This was an important element relating to the personal meaning of this project, the knowledge that I could make a contribution based on my expertise as a researcher. As a matter of fact, I alluded to my commitment and strong desire to contribute to what was happening in PR, in all my emails, phone conversations, and in-person meetings with community leaders, when discussing the study and asking for help with finding potential participants.

### Inception of the study and considering context

My colleague, Dr. Engelman, has done significant work in disaster research. The severity of the subject matter gains significance if one considers that, despite more initiatives to include people with functional diversity or access needs and older adults in emergency management, the response and recovery efforts continue to be left out of the narrative of emergency preparedness, while the likelihood of future climatic catastrophes is always increasing (Engelman et al., 2013; Ivey et al., 2014).

As we began to design the study, we considered how the toll in recent years of economic recession, and a neglectful and exploitative colonial relationship with the US, had exacerbated the devastation caused by the hurricane. After several years of fiscal hardship, the local government acknowledged in 2015 that it would be unable to pay an insurmountable debt, which was related to a lack of administrative funds, illegal lending practices, and budget mismanagement (Meng, 2019). In 2016 a new law was enacted, the Puerto Rico Oversight, Management and Economic Stability Act, or PROMESA (‘promise’ in Spanish), which empowered the US President to appoint a seven-member Financial Oversight and Management Board (FOMB) that has ultimate control over PR’s budget and would oversee the debt restructuring. Most residents of PR were indignant about their local government’s reduced autonomy. The unelected members of the Board exerted their control by increasing taxes while curtailing public services and reducing workers’ benefits, which continues to date. PR, at the time, was considered poorer than the poorest US state and was dealing with severe austerity measures imposed by the FOMB.

This is the scenario that prefaced the category-5 Hurricane that struck PR in September of 2017. Hurricane María brought devastating effects that were only aggravated by the previous conditions in PR (Bonilla & LeBrón, 2019; Sullivan, 2018). A study led by researchers from Harvard T.H. Chan School of Public Health (Kishore et al., 2018) that examined the number of excess deaths after Hurricane María, found that there was a 62% increase in the number of deaths in the three months following the hurricane, with a total estimate of 4645. A study by Cruz-Cano and Mead (2019) found that among the number of excess deaths in their own study, all were older adults over 60, with some of the causes including heart disease (the most common), followed by diabetes, Alzheimer’s disease, and septicemia. These conditions likely worsened because of the limited health care services and damaged infrastructure (Dreisbach, 2019; Minet, 2019; Roman, 2017) impeding people from obtaining the adequate treatment. All these pre- and post-hurricane contextual issues informed our thinking and the design of the study.

As we assembled our research team, there was an intentional focus on including academics claiming various identities, as disabled and as Puerto Rican, academics focusing on qualitative research, as well as researchers living in PR who had done work in poor communities there. Alina Engelman and I were the first to begin planning the project, and we used a mixed-methods approach, with the quantitative methods guided by Engelman’s previous work (Engelman et al., 2013; Ivey et al., 2014) on a CDC-funded nationwide emergency preparedness project at UC Berkeley. For this paper, we focus solely on the qualitative component of the full study. Soon after Alina and I started working together, Irina Todorova (Northeastern University), who has extensive experience in qualitative research in the context of health inequalities, including older adults, joined the team. Additionally, we sought the collaboration of researchers living in PR, who were embedded in the same circumstances as other Puerto Ricans, and who could understand more than any of us on the US Mainland what it was like to be in pre- and post-María PR. Scholars living and working in PR, Rosa Guzzardo Tamargo, Evelyn Dean-Olmsted, and Alicia Rivero-Vergne, joined the research team to ensure that our design and approach would safeguard cultural humility, and for support with participant recruitment. In order to conduct effective research, it is highly beneficial for the composition of the research team to reflect the community under study. As a linguist and a linguistic anthropologist, respectively, Guzzardo Tamargo and Dean-Olmsted encouraged awareness of the context-dependent and multidimensional nature of linguistic expression (e.g. involving gesture, posture, tone, gaze, etc.), while Rivero-Vergne contributed expertise in qualitative design and research within impoverished communities in PR. She was consulted on the interview guide to ensure that it was culturally and linguistically appropriate and that participants would not have trouble understanding the questions.

Our discussions led us to select Interpretative Phenomenological Analysis (IPA; J. Smith & Osborn, 2003) as the qualitative methodology for our study. This decision was motivated by our realization that the experiences of older adults (65 or over) with functional diversity, living through an intense disaster are not well understood, and this information would be important for improving future support. The older adults who participated in the study had a functional or access need that affected some form of daily living (i.e. activities of daily living and instrumental activities of daily living). Thus, we took an idiographic approach, with a focus on how individuals went through the hurricane and its aftermath and how they perceived the meaning of this experience, based on each person’s unique perspective and context. We chose IPA since it ‘allows a valuing of agentic individual subjectivities and voices otherwise ignored or silenced’ (Todorova, 2011). At the same time, while an idiographic approach is usually seen as an in-depth focus on individual experiences, from the beginning, we were certainly aware of the complexity of the socio-cultural and political context in which this disaster, and, thus, our study, were taking place. We expected that our methodology could potentially undergo shifts during the study, to understand ‘the person-in-context’. IPA has developed in diverse directions since its establishment as a coherent methodology two decades ago (J. Smith, 2011), some of which have explicitly reflected on what it means to expand the interpretative and contextualizing potential of IPA (Larkin, Watts, & Clifton, 2006; Todorova, 2011). A phenomenological approach to understanding experiences related to a disaster of this magnitude needs to reflect on how to integrate the socio-cultural situatedness of these experiences, including the relevance of social exclusion and inequalities (Clifford, Craig, & McCourt, 2018), and we believe our work contributes to this reflection.

## Method

### Reaching participants: community leaders as gatekeepers

In June 2018 (seven months after the hurricane), I was in PR with the goal of collecting data, and the task to find participants felt daunting. Fortunately, my mother, who is a member of feminist groups in PR, put me in contact with local activists, academic activists, and individuals who took the lead in local recovery efforts. These individuals shared their emails and phone numbers with me, and I proceeded to write to, and call, as many people as I could with a description of the project and the characteristics of the participation requirements. As I continued to recruit participants, I became more and more concerned about how the study would be perceived by the people there. Puerto Ricans had just been through a major disaster, and there were discussions on social media about how US Americans were suddenly interested solely for self-promotional objectives, with images of people taking pictures of themselves collecting or delivering supplies. I was afraid of being perceived as someone taking advantage of a humanitarian crisis. For this reason, in my emails and phone conversations about the study, I provided some information about myself, explaining that I grew up in PR and felt anguish about what was happening, and that I wanted to do something related to my expertise that would be helpful in order to increase awareness in the US about the situation of older people with access and functional needs in the aftermath of the hurricane. I believed that explaining my connection to PR would give me some validity and help me be perceived as worthy of this undertaking. In this case, being honest about my own subjectivity was essential to making connections in the field.

Most of the individuals I contacted received me with open arms, although some ignored my calls or emails. Communication was still very difficult in the more mountainous regions, but many of the leaders in the recovery process were traveling frequently to the capital for supplies, and it was then that they were able to answer my emails or calls. Some interviews also took place with family members of friends. Therefore, a combination of snowball and purposive sampling techniques were used in participant recruitment.

I drove to each of the interview locations (some near the capital and most over 90 min away within the mountainous range along the center of the main island of Puerto Rico), and I was accompanied by either one of my sisters or a colleague on the project, who served as notetakers. It was important to have a notetaker at each of the interviews, capable of capturing gestures by the participants, nonverbal cues, as well as jotting down information on the reasons for interruptions during the interview, side-note observations about participants, and comments on the context. The importance of notetakers and of seizing features about the context cannot be underestimated. During this process, I realized the value of not only capturing the words of the interviews, but also the context in which the interviews took place. The role of assistants was key in this respect, contrary to the common imagining of ethnographic research as undertaken by a ‘lone wolf’ fieldworker. For anyone conducting disaster research, taking notes and pictures, and recording conversations with others before or after the more formal interviews, is crucial to attaining a vivid picture of the study’s focus, and helps to understand the multiple dimensions of the data from interviews with survivors of a disaster. In ethnographic analysis, everything around you becomes your research, and I wished I had accounted for that in the protocol that I submitted for the ethical approval of my study to the Institutional Review Board at CSUEB. Given that I left it out, when reporting or presenting the findings, I could not include my conversations with activists about their personal experiences during and after the hurricane as data. Yet, I was able to use those experiences in the field to expand the IRB protocol and inform my interpretations of the findings (which is routinely done in ethnographic work, e.g. Pinsky, 2015).

Previous discussions with activists were also essential to understand the context of the interviews. In Orocovis, located in the Central Mountain Range, I met a local community organizer, who led me to various homes whose residents were interested in being interviewed voluntarily. After I had emailed her, she took it upon herself to find people who would be willing to participate, and I am eternally grateful for her kindness and support. She, as well as other activists and community leaders, were instrumental in helping me reach potential participants. This community organizer met me at the local church where I was able to converse with women from the church and with a group of nurses in a community organization whose mission was to offer health education and to provide care to community members. They discussed how people of Orocovis were faring after the hurricane, the needs of the community and the particular challenges of living in this mountainous region after a disaster of such magnitude. I was already informed regarding this situation because the news reports had discussed the difficulty of getting supplies to these areas due to blocked roads. They had emphasized how older people’s health was not being maintained because of a strained medical system after the hurricane.

At the same time, on a more personal level, it was breathtaking to simultaneously absorb the scenery. Despite living in PR throughout my childhood and into young adulthood as a college student, I had never been to this part of PR before. The church sat on top of a hill that overlooked the continuous stretch of mountains where the greenery, which was coming back after being wiped out, seemed to be speckled with dots of white roof tops and blue tarps (roofs covered in blue tarp from FEMA). During my drive to Orocovis and other parts of PR, I had noted the bare trunks of the trees on the side of the highway. The scenery down these country roads, which is usually replete with luscious greenery, was still bare of leaves and riddled with exposed tree trunks, an unfamiliar and foreboding site for anyone accustomed to the PR landscape. However, from this hill-top church, seeing how some green was coming back, and, at the same time, witnessing how these women cared for older people seeking medical attention, provided motivation and hope.

The same beautiful views could be appreciated from la Loma de la Niña Mariana in Humacao and served as a backdrop to the strength of the community’s organization, which was nothing short of inspirational. A community organization that was initially dedicated to celebrating breadfruit (*pana*), it evolved into a collaborative, grass-roots endeavor that fed hundreds of neighbors and workers helping with the recovery. The local community organizers led me to where the people of this town met, at a hill-top that had a structure with a kitchen. There were many people there who had congregated to make plans and to cook for each other as well as for others. This organization served a life-sustaining purpose for the community and it was electrifying to see it at work.

### Insider/ outsider positioning

As I began interviews in participants’ homes, I introduced myself again, depending on my insider connection to PR to gain their trust. My background and having family members in San Juan who had lived through the hurricane themselves were important. At the same time, I presented my outsider position, coming back after the hurricane, wanting to contribute, with this study, to the understanding of what people were going through. For the most part, participants were forthcoming about their experiences, which were still very raw, making these interviews the most emotional and tearful I had ever conducted. Additionally, some seemed angrier and more indignant because of not receiving enough government support after the hurricane. These are moments in which one must let their emotions come through and not seem agitated or upset, but rather, demonstrate quiet acceptance so that participants can express themselves. I cannot say I was always able to achieve this, but I believe it must be a goal in interviews with survivors of disasters.

I also had to explain repeatedly that I was not a journalist because some of them had been interviewed by local and US Mainland news media. When conducting this type of study, it is necessary to explain the purpose of the research and have a brief description of the goal of the interview. In many cases, I had to insist on longer responses to my questions through prompting their answers or probing for elaboration. This is the challenge all qualitative researchers face, but, in this particular situation, part of the reason why participants provided short answers was because of their recent experiences with quick question-and-answer sessions with journalists, different from the detailed and elaborated responses that are characteristic of in-depth interviews like the ones I was conducting.

While I did try to introduce myself as having a background in PR as a way of underscoring a shared cultural background with my participants, I acknowledge that this may be unrealistic. For example, there are so many positionalities to negotiate and the way that I am perceived by the individual, a part from who I feel that I am, is also consequential. For example, those in more rural areas may perceive me as an outsider when I explain that I grew up in the metropolitan area, given the social and economic perceived differences between these groups (e.g. those in the mountainous/ rural areas are perceived as of lower social class or uneducated while those in the metropolitan area are perceived as possibly more Americanized, superficial, or even dangerous). Given these many nuances about various mixing positions and identities, the very idea of being an ‘insider’ investigator is problematic. Narayan (1993) questions the authenticity of any insider status, and eloquently discusses the essentializing nature of the label:

The loci along which we are aligned with or set apart from those whom we study are multiple and in flux. (…) what we must focus our attention on is the quality of relations with the people we seek to represent in our texts: are they viewed as mere fodder for professional self-serving statements about a generalized Other, or are they accepted as subjects with voices, views and dilemmas- people to whom we are bonded through ties of reciprocity and who may even be critical of our professional enterprise? (p. 672)

Therefore, when thinking about positioning ourselves (insider/ outsider) for our own research purposes, we must first give equal, if not more, weight to the goals of those participating in the research and also acknowledge that the distinction between insider and outsider positionalities is quite blurred and indefinable.

### Feelings of outsider identity in relation to the deaf community

Although I was able to connect with many people on the basis of our shared concern for PR, the participant-researcher connection was not achieved in all cases. My attempt to reach the deaf community in PR, as a hearing person who had never conducted research in this community, was a real setback. Reflecting on this experience illustrates well how a researcher’s goals can be stymied without previous, in-depth knowledge of the community in question. While I have done research with older adults with access and functional needs, working with the deaf community was completely new to me. I thank Dr. Alina Engelman, a deaf academic on our team, for opening my eyes to the experiences of a group of people that is largely ignored in PR (and on the US Mainland as well), and not included in the narrative of the news media in PR. Unfortunately, she could not be with me for the participant recruitment and data collection. Through a community organizer, I was able to reach two deaf women from an impoverished, remote area of PR. They wanted to be interviewed at the same time. One of them was younger than 65, but insisted on being interviewed. When I attempted to suggest that I could not interview someone under 65, they seemed to get upset (frustrated and saddened). Consequently, I interviewed them together as they wished, being flexible with a planned method in response to the specific needs of the participants and the context. They lived in a community of deaf people who have a hereditary deaf condition. As I drove in, I was able to observe many groups of deaf people walking up and down the hill; the activists and community leaders taking me to the deaf women’s homes explained that most deaf persons in the community did not drive and only got around by walking. It is sobering to think about what this community endured after the hurricane. I discovered that some hearing individuals are prejudiced and annoyed with this community. Furthermore, they apply racist nicknames to them because they tend to have a darker skin complexion. Issues of ableism and racism intersect in a profound way in this area of PR.

Another issue that I encountered was that the interpreter, who was a hearing friend of people in this community, tended to answer for the participants. I had to continually ask her to let the participants answer for themselves, for which she seemed to take some offense, as if thinking that I did not believe her. Consequently, despite my explanations, there was a miscommunication and lack of understanding regarding the reasons for needing to compile their experiences from the perspective of the deaf individuals. The need to find a professional interpreter who either was not so embedded in the community or adhered to professional standards (the Code of Professional Conduct outlined by the Registry of Interpreters for the Deaf and required for certification) would have been more appropriate. However, at the same time, the participants expressed the desire to have her as their interpreter and may have not participated in the interview without her. These interviews were set up by a community organizer who waited outside of the home while I conducted the interviews. It would have been more valuable to gain a better understanding from the interviewees, but they were reluctant to say much. Instead of being able to balance a position of outsider/ insider, as I did with the other participants, with these women I felt that I was only an outsider. There was less possibility for me to build rapport with them and I felt a disconnect throughout the entire interview. The community organizer, who seemed sympathetic toward them (who took me to the house where they were), also gossiped and whispered to me as we left about sensitive and private issues regarding the participants. This happened in front of the participants. I was worried that they could read our lips or sense that we were talking about them. It was impossible to negotiate elements of insider status with the Deaf individuals within the limited time frame available to me.

I also wanted to include perspectives of deaf individuals in San Juan, the PR capital and its largest city. I knew they met on a weekly basis in a café and decided to go there. Again, I felt completely out of place. I had never been in a position of minority status with the deaf community. There was an interpreter with me, who I found through other community activists. I prepared flyers about the study in Spanish with the purpose of going to the café to recruit participants. The interpreter sent them back to me before our plan to meet because I had written ‘people with disabilities’ and she said that the deaf community in San Juan prefers to be described as ‘people with functional diversity.’ When we arrived at the café, the older deaf individuals could not read the flyers because they were in Spanish. The interpreter and I learned that the older generations of deaf people in PR were taught written language in English and could not read in Spanish. All this reveals my own lack of knowledge about this community and the need to be better prepared before immersing myself in this subject and attempting to interview individuals from this community. It felt uncomfortable to be at the café, and, again, I could not build rapport and felt like an outsider; moreover, I did not feel free to discuss my study with the people there. Finally, I also felt uncomfortable because I needed to have an interpreter rather than being able to use sign language myself. In this café, as I entered this deaf community’s social space I became ‘the minority’ and one could view this as an imbalance of power. This contrasts with most scenarios in which the researcher can be considered the one with more power (as discussed earlier based on Bourdieu’s description of the social asymmetry in the research relationship). However, even though it may seem like I have less power within the café (as a hearing person within a social space for deaf individuals), I am still entering this space to pursue my own research agenda, which can be considered an imposition. Additionally, this café is located within a world that devalues and problematizes being deaf. I have only to step outside to reenter my privileged state in our society. Therefore, even in this scenario in the café amongst deaf individuals there still may be a power differential between researcher and potential participants, which one must consider, critically examine, and attempt to reduce or ideally eliminate – one of the goals of decolonizing methods (Keikelame & Swartz, 2019; Tuhiwai Smith, 2012).

Being Puerto Rican or having family who went through the hurricane were not sufficient factors for me to obtain insider status nor build sufficient rapport with some participants. Tuhiwai Smith (2012) discusses the particular need for insider research (when one identifies with the community being investigated), arguing that the research ‘has to be as ethical and respectful, as reflexive and critical, as outsider research. (…) It needs to be humble because the researcher belongs to the community as a member with a different set of roles and relationships, status and position’ (p. 140). The reflective approach enables me to examine my various positions and how they influence my interpretation of participants’ experiences. It also puts into focus the limits of those positions as well as the insider status one may think to have. Indah (2018) suggests that one cannot depend on insider status based on a single attribute. For example, in my case, with respect to the older adults, having lived in PR and feeling a sense of solidarity from that shared trauma was not enough. Also, regarding the case of the deaf individuals, I was further distanced from their experience given their specific challenges of surviving the hurricane and its aftermath.

### A transformable space: relational experiences Makes research come ‘Alive’

Initially, I expected the interviews would be conducted one-on-one, as is typical for most qualitative interview methods and originally saw the presence of other people as interruptions. One participant lived with his sister, who entered the house during the middle of the interview, back from the grocery store, in the company of a friend. I became very worried about the lack of confidentiality for the participant because I had envisioned most interviews with no one else present, except the third party notetaker. However, with this first interview, and several that followed, a process of relational inquiry emerged as I realized that interruptions were not only unavoidable, but, instead, integral to the process (Newbury & Hoskins, 2010; Younas, 2017). Sometimes the participant would insist on continuing the interview with other people present, such as in the case of this man, whose sister entered the home during the interview; he insisted that I continue with the interview in the presence of his sister and her friend. However, he seemed to change with their presence (aware of their presence) and become more reticent to discuss certain experiences. In other cases, participants insisted that their adult children be present during the interview and sometimes these individuals responded for their parents despite my gentle plea that they let their parents answer for themselves. I questioned whether it was right for me to do this, given that the older adult may prefer that their children contribute to the responses. Challenges surface when adult children answer for the older adults. While qualitative researchers aim to conduct interviews in the most comfortable manner for participants, we take into account that what is shared during the interview in the presence of others is different from what would have been shared individually. Within gerontological research on interviewing older adults, scholars acknowledge that sometimes older adults feel more comfortable with adult children present, even though one must realize that older adults will be less candid in their children's presence (Gerolimatos, Gregg, & Edelstein, 2014). There are evident concerns for maintaining standards of confidentiality when another person enters the interview setting, which point to needing to reconsider the meaning of ‘confidentiality’ within the specific interview relationships.

I could not ask others to leave because I knew that their presence was helping their elder parent feel more comfortable with me. At the same time, I accepted that the stories would be different than what they would have been without a family member present. Nonetheless, I would not have been able to interview them otherwise. The approach was fluid and interactive; rather than focusing on the ‘correct’ method and holding on to a predetermined idea of what it entails, the unique situations researchers face in the field catalyze the creative application or combination of methods as well as the development of new ones (Chamberlain, 2000).

This relational part of the inquiry that became transformational was also evident when I was led to the hill-top community gathering place that I previously mentioned, in Barrio Mariana, of Humacao. When I arrived, I realized that there were potential participants who wanted to be interviewed in a corner of this space, outside, under a gazebo, in front of the numerous people there surrounding us, going about their own business, either casually conversing, eating or discussing organizational issues. I was concerned about maintaining their confidentiality and with the lack of privacy (I had planned on only interviewing participants in their own homes). However, the potential participants in this place insisted that they be interviewed there. This was a space that they cherished, where they felt comfortable, with people they ‘loved’ who were ‘like family,’ in their words. Something similar occurred with the individuals with hearing impairments from Miraflores, Orocovis, with whom I adapted to having a younger person interviewed, along with the older person, given that the elder participants seemed to prefer it. Again, in these examples, methodology is transformed to fit with the preferences and needs of the research participants.

### Flexibility and sensitivity

The interview questions were semi-structured prompts so the interviews did not always follow the chronological order of the interview guide which aimed to reconstruct the story of before, during and after the hurricane. When asking the first question about expectations, many participants tended to dive into their experiences in the aftermath. After all, these were the most recent experiences and, also, the most urgent to be communicated, given the evident level of need that was still apparent in their communities and in PR in general. I had trouble moving away from the order of the interview guide for fear of not covering some questions about a particular period of time. In some cases, I unfortunately did interrupt participants to pull them back to the time before the hurricane, and when I returned to the time after the hurricane, they sometimes were less emotive, and I had to probe about something they had said earlier. At that point, sometimes they were not as interested in continuing that line of conversation, and I realized that I should have stayed with the flow of their own thinking. In fact, it was more important for me to follow their lead and let participants talk about the experiences in the order that they preferred, especially given the IPA focus.

## Discussion

IPA can contribute to our understanding of a particular experience from the perspective of the individual (idiographic approach), with a consideration of the meanings that individuals ascribe to those experiences. An important element of IPA is reflexivity, because in the process of examining the subjective experiences of others, the researcher must be ‘mindful of their own beliefs, perceptions, and experiences so that they can enrich their interpretations rather than them being an obstacle to making sense of the participant’s experiences’ (Peat, Rodriguez, & Smith, 2019, p. 8). This is achieved through reflexivity at the beginning, while designing the project, in the process of conducting the research, and during the formulation of interpretations based on the analysis. Peat et al. (2019) add that reflexivity also occurs *a posteriori*, after the research concludes, and it comprises the ways in which the research encounters impact both the researched participants and the researcher. This develops into a ‘hermeneutic circle.’ The different positionalities (e.g. preexisting roles, identities, level of familiarity between the researched and the researcher) of the researcher may impact the participants’ articulation of the experience as well as the researcher’s interpretation of their articulation. This encompassing reflexivity enriches one’s understanding of the data and supports a critical examination of the research experience.

We followed the principle of reflectivity and, moreover, in the post disaster context of Hurricane María’s effect on PR, our method transitioned into ‘working pluralistically’ and integrating considerations from different approaches (Chamberlain, Cain, Sheridan, & Dupuis, 2011). For instance, more relational inquiry was incorporated into the study’s implementation. Relational inquiry (with philosophical underpinnings similar to those of IPA, including pragmatism, hermeneutic phenomenology, and critical theory) explores lived experiences in an intersubjective way, considering the influence of the participant-researcher relationship and interaction (Held, 2019; Younas, 2017). Another aspect of relational inquiry is the intersubjectivity of the lived experience that is studied, related to how other people in the individual’s life affected that lived experience and that person’s perception of their experience (Newbury & Hoskins, 2010). Consequently, it was necessary to have family members take part in the interview when participants requested their presence, and to not do so when they suggested otherwise. This was also the case in some participants’ preferences to have interviews in public spaces among their neighbors (a situation that made the participants more comfortable), and by reaching participants through community leaders in the response and recovery effort. The philosophical underpinnings of IPA and relational inquiry can go hand in hand. In fact, the element of intersubjectivity of the experience, evoked through relational inquiry, can add depth to the analysis.

A crucial element of decolonizing methodology incorporates participants’ perspective in the research (Tuhiwai Smith, 2012), as occurs with community-based participatory research, encouraging the self-determination of the community involved in the research. According to this methodological approach, participants share in the study’s design, and the findings can be used to transform an oppressive or unjust context (Held, 2019). A decolonizing framework is particularly relevant for research in PR by individuals who live on the US Mainland because it alters the traditional power relations between the participants and the researcher. Rather than taking a settler/ colonizer view of the research activity, that is, one that upholds a power dynamic with the researcher as the know-er and decider of how research is conducted and how data are interpreted, decolonizing methods alter this hierarchical dynamic. In this study, we were open to having the participants in the project make decisions about how the research should be carried out (with interruptions, with family members present, with their choice of interpreter, in the place of their choosing, etc.; Keikelame & Swartz, 2019; Tuhiwai Smith, 2012; Zavala, 2013). Creating space to transform predesigned research when we are in the community conducting research creates opportunity for meaningful research encounters. In anthropology, a flexible approach to research that can accommodate the ‘messiness’ of reality on the ground, coupled with continuous scrutiny of how researchers’ positionality within power structures influences their perspectives and interactions, has been standard since the field’s so-called ‘reflexive-turn’ beginning in the 1980s (associated with authors such as Clifford & Marcus, 1984; Geertz, 1988; and Behar, 1990, etc.). Among the limitations we acknowledge is that it would have been beneficial to also consult with interviewees themselves on the interpretation of the findings; however, this was not articulated in the institutional review protocol. In our case, we did consult Puerto Rican scholars and local activists who experienced the hurricane themselves and its aftermath while living in PR, on the design, formulation of questions, and the interpretation of the findings. In the future, we would consider more involvement by participants (or ‘co-researchers,’ Finlay, 2009) on the design and interpretation of the findings, as is done in studies using community-based participatory research or decolonizing methods, and we encourage future disaster researchers to continue to do this. When researchers integrate participants giving them a decision-making role in how they will participate in the research, it creates space for transforming methods, an approach that is akin to the intent of decolonizing methodologies, in which research participants are not the object of research, and, instead, ‘questions are framed differently, priorities are ranked differently, problems are defined differently, and people participate on different terms’ (p. 196).

With more researchers on the US Mainland conducting studies in PR, particularly because Hurricane María shone a spotlight on PR, there must be an awareness of, and a sensitivity to, the exploitative and hierarchical relationship between the US and PR. Moreover, there must be a mindfulness of how Puerto Ricans perceive themselves in relation to individuals from the US Mainland who conduct research there. There is a traumatic colonial legacy involving the abuse of power and exploitation (Morales, 2019). This is particularly evidenced through the historical abuses of US American researchers and health care professionals toward Puerto Ricans (forced sterilization [Briggs, 1998], possible medical torture [Starr, 2003], and use as test subjects for experimental contraceptives [Verma Liao, 2012]). One could argue that the reasons why decolonial methods are necessary with research related to Indigenous people are similar to those why decolonial methods are urgent when US American researchers study the experiences of Puerto Ricans in PR. These groups face subordination, marginalization, and exploitation by the US government, as well as a loss of control of their land at the hand of the US government or corporations from the US private sector. As a researcher coming from the US Mainland, one must be sensitive to issues of colonial repression, the power dynamic between the US and PR, and the inequity with which Puerto Ricans live, as compared to individuals living on the US Mainland.

Incorporating ideas behind decolonizing methods can ward off exploitation, by deconstructing notions of best practices that align with positivist principles. Following more positivist and deductive approaches with an emphasis on objectivity and neutrality, creates a separation between researcher and participants, with the idea that the researcher has ‘the skills’ to perceive that which is somehow imperceptible to the participants in the research study. Sometimes, research such as the study described here, which *must* be relational, consciously subjective and capable of reflexivity and critique, might be devalued. For example, Tuhiwai Smith (2012) explains that ‘institutions such as the academy and major funding agencies maintain and reinforce the idea that research is a highly specialized skill which by definition ‘has to be’ developed and supported at a distance from the community’ (p. 128). However, it is necessary to have the participants and the researcher work together to understand the lived experience being explored. Decolonizing methods encourage appreciation for more embodied, affective, relational ways of being and knowing, which necessitates flexibility, humility, and reflexivity in research (Nicholls, 2009). Finally, it is probable that in collectivist cultures, such as that of PR, in which the goals of the group carry more weight than individual goals, the intersubjective and relational quality of the experience is more meaningful.

Given that PR has been considered a collectivist culture (Gudykunst & Ting-Toomey, 1988) – even though there is a lack of recent empirical evidence to support this –, there may be a need for relational methods when conducting research there, which allows families to be involved in the interview, permits connections and involvement of community leaders, educators and activists, and welcomes the warmth of community activists and leaders when offering food, coffee, gifts in gratitude, etc. One can presume that the meaning of the experience for the individual relates to the very fact that it is a shared experience.

### Suggestions for future post-disaster qualitative research (in Puerto Rico and Beyond)

Collaborators on this project have identities that entail similar attributes to those of the individuals participating in the study. Collaborators also come from a variety of disciplines to provide an interdisciplinary focus to the research. The diversity of the research team contributes some level of awareness of the issues that may be confronted by the participants. Nevertheless, Tuhiwai Smith, in her discussion of decolonizing methods, cautions against using one’s identity or connection to the community as a way of claiming expertise or understanding of the study participants’ experiences. As mentioned previously, a humble and respectful approach is necessary and one cannot assume that insider status is a gateway or facilitator to understanding participants’ experiences.

Future studies would best incorporate community-based participatory research which is embedded within a decolonizing framework, emphasizing the need to work within the community in all aspects of the project, from its inception to reporting the findings and the use of the results to benefit the community. Tuhiwai Smith lists the important qualities about the ‘process’ of community-based approaches, including having respect, enabling people, creating opportunities to heal and to educate, and contributing to self-determination (p. 130).

Relational inquiry emphasizes the intersubjectivity of the experience, not only pertaining to the researcher/participant relationship in the research process, but also in regard to the fact that the trauma associated with a disaster is often a collective experience, and meaning is attributed through this shared trauma. For example, rather than having an individual interview, having family members engage with the participants in the recollection and articulation of experiences and events that occurred, may sometimes be the most appropriate course of action. Community-based participatory research (Gibbs et al., 2018; Wallerstein & Duran, 2010) participatory health research (Abma et al., 2017; Groot et al., 2019) and the decolonizing framework (Tuhiwai Smith, 2012) are examples of methods that allow for relational approaches. Similarly, relational phenomenological designs that include participants in the research process should consider the participants as ‘co-researchers,’ ‘retaining an open, empathic, embodied presence to another’s personhood, given the position that what we can learn and know about another arises within the intersubjective space between’ (Finlay, 2009). This ‘in between’ space is challenging to manage within the research context, but it is crucial in order to understand, in a truly empathetic way, the experiences being investigated.

The imperative of reflexivity needs to be a constant in research. It begins with reflecting on our own preconceptions, behaviors, biases, and beliefs when designing the research or carrying it out, but it does not end with the conclusion of the study. Rather, it continues into a process of meta-reflexivity, in which we reflect on our own reflexivity, as we have done in this article. An integrative element of this meta-reflexivity is humility. As we step into the researcher role to seek answers to our inquiries on issues of vital importance to the communities that we study, we must assume a humble stance that is open to self-critique. This is particularly important for research with older adults and people with access and functional needs in the post-disaster context. These are individuals that are often neglected, treated with condescension, and whose autonomy is constantly being threatened by others.

The researcher’s humility when working with older adults or people with access and functional needs who have lived through a disaster must entail creating space for transformable methodology (i.e. flexibility, adaptability), in order to accommodate for situational circumstances as well as the needs and preferences of participants in a post-disaster context. This would take into consideration ‘human subject’ ethics approval, so applications to internal review boards and ethics committees would ideally include possibilities for broader ethnographic methods. When methodologies change or mutate, they serve the transformative purpose that breaks with existing paradigms. This transformable space is part of the research process within a decolonizing framework, in which the researcher and co-researchers (i.e. participants) may use the original design creatively, such as not adhering to the questions in an interview guide, or having the research take place within the most comfortable setting for participants in a context of their choosing (e.g. with family around, participating in the interview, while having coffee, within a public communal place in the presence of neighbors, or alone in the privacy of their own home). In the case of using the interview guide flexibly, this would include a dialogical balance between (1) a researcher’s interest in looking for patterns within the participants responses (which is one of the most common goals in qualitative analysis) along with (2) the goal of understanding participants’ unique experiences. Overall, changes to the original design, within the context of qualitative disaster research, should not be perceived as a researcher’s lack of skill or a hindrance to the project, but rather this transformability should be seen as necessary, appropriate, and even ethical.

While many qualitative researchers embrace and acknowledge the subjective and relational quality of their research, it is not always perceived as scientifically sound. Decolonizing methods delegitimize ideals of objectivity or neutrality within any type of research which maintain power dynamics that marginalize participants in a study. Instead, they seek the same type of emancipatory perspective that encourages participants’ self-determination (Tuhiwai Smith, 2012). Through a decolonizing framework, a qualitative researcher can reduce the power differential that frequently exists between the researcher and the participant (e.g. as previously described in the experiences related to insider/ outsider positionalities). The power differential is also apparent in the context of a researcher from the colonizer land, trained in the colonizer research paradigms, coming into the colonized territory to conduct their own individually developed research. When speaking about indigenous people, Tuhiwai Smith explains that colonization comes with imposing a ‘positional superiority over knowledge, language and culture’ (2012, p. 67). Parallels can be drawn given the background on PR discussed previously, considering what has occurred throughout the history of the PR territory (e.g. through control of the school system, imposition of English language, and the prohibition of nationalistic symbols, and also currently, through a manipulation of information such as the federal government’s questioning of the death toll after the hurricane or the level of mismanagement of the response). This results in the potential imposition of knowledge, culture, and presuppositions that come with a research design. Reflexivity and a decolonizing framework create an opportunity to avoid or reduce this imposition.

One must also be open to reconceptualizing researcher positionality, reflexivity, and ethical practice within the context of loss and suffering that may emerge after a disaster. Guillemin and Gillam (2004) discuss the importance of ethical reflexivity when interviewing individuals who are discussing distressing experiences: ‘reflexivity does not prescribe specific types of responses to research situations; rather, it is a sensitizing notion that can enable ethical practice to occur in the complexity and richness of social research’ (p. 278). Along the same lines, Bowtell et al. (2013) explain that in reflecting on, and sharing, the emotional and ethical challenges encountered in research, we can learn from our experiences and encourage a rich dialogue about qualitative disaster research. In the post-disaster context of Hurricane María’s effect on PR, our method included welcoming a transition into ‘working pluralistically’ and integrating considerations from different approaches (Chamberlain et al., 2011).

Intersecting identities among participants as a Puerto Rican and a functionally diverse person brings a new dimension to decolonizing the oppressive underpinnings of research when it is considered ‘the domain of experts,’ objective truth seekers, with institutionally valued qualifications and skills (Tuhiwai Smith, 2012, p. 127) . This kind of research may harmfully and mistakenly attribute the locus of the problem on the Puerto Rican community. In the case of the present study, the disaster and humanitarian crisis after the hurricane could be seen as an inherent problem of the Puerto Rican community or as a result of Puerto Ricans’ own actions, rather than because of structural and social context (i.e. the colonized condition of PR). Therefore, research must be centered on the researched community’s perspectives, values and goals (Tuhiwai Smith, 2012). Furthermore, incorporating functionally diverse populations into decolonizing research supports the questioning of the Western notion of ‘disability’ as abnormal or as a physiological problem, and, instead, considers that the problem lies with society and our constructed environments which disabled individuals (Russell & Malhotra, 2019). These views lead us to reject a normalization of certain abilities or dependencies over others in a post-disaster context, leading to more inclusive emergency planning and management.

By following a decolonizing framework, any qualitative research on individuals’ experiences surrounding a disaster would use the findings in such a way that it improves the lives of the individuals involved in the study and their communities. This is why activism is a part of the decolonizing framework, in which researchers transform the findings into action-oriented goals that revolve around promoting social justice within the communities they have worked (Braun et al., 2014; Keikelame & Swartz, 2019; Tuhiwai Smith, 2012; Zavala, 2013). We will continue to discuss our findings within PR and the US Mainland in order to argue for improved disaster preparedness and related justice in the response and recovery efforts of local and federal governments, with a clear focus on older adults and individuals with functional and access needs.

## Notes

1 The first person in this article refers to the first author, who did most of the ethnographic work. Later in the article, ‘we’ is used for the entire research team, which collaborated on project design, data collection, data analysis, the preparation of presentations on findings, as well as the preparation of this manuscript.

2 ‘Functional diversity,’ rather than ‘people with disabilities,’ is a self-described term preferred by some of our participants. Emergency management officials have increasingly begun to use the term ‘access and functional needs’ to refer to people with disabilities in a disaster context. We use ‘functional diversity’ and ‘access and functional needs’ interchangeably.

3 Puerto Ricans have US citizenship, but cannot vote for the US President in the general elections (unless they are residing on the US Mainland), and they lack voting representation in US Congress. The people residing on the islands of the archipelago elect their local government officials and up until recently (discussed further below) had much of the control over local affairs, while still subject to any federal regulatory restrictions.

4 This panel discussion occurred prior to the recent protests of Summer 2019 in PR to oust the governor, which surpassed the number of people who participated in the protest against the marine base in Vieques.
